# SARS-CoV-2-Vaccine-Related Endocrine Disorders: An Updated Narrative Review

**DOI:** 10.3390/vaccines12070750

**Published:** 2024-07-08

**Authors:** Avraham Ishay, Kira Oleinikov, Elena Chertok Shacham

**Affiliations:** 1 Endocrinology Unit, HaEmek Medical Center, Yitzhak Rabin Av. 21, Afula 18101, Israel; 2 Faculty of Medicine, Technion-Israel Institute of Technology, Haifa 31096, Israel

**Keywords:** COVID-19, COVID-19 vaccines, SARS-CoV-2 vaccine, endocrine adverse events, thyroid, pituitary, adrenal, diabetes, gonads

## Abstract

The emergence of the COVID-19 pandemic has led to the rapid and worldwide development and investigation of multiple vaccines. While most side effects of these vaccines are mild and transient, potentially severe adverse events may occur and involve the endocrine system. This narrative review aimed to explore the current knowledge on potential adverse endocrine effects following COVID-19 vaccination, with thyroid disorders being the most common. Data about pituitary, adrenal, diabetes, and gonadal events are also reviewed. This review also provides a comprehensive understanding of the pathogenesis of endocrine disorders associated with SARS-CoV-2 vaccines. PubMed/MEDLINE, Embase database (Elsevier), and Google Scholar searches were performed. Case reports, case series, original studies, and reviews written in English and published online up to 31 August 2023 were selected and reviewed. Data on endocrine adverse events of SARS-CoV-2 vaccines are accumulating. However, their causal relationship with COVID-19 vaccines is not strong enough to make a definite conclusion, and further studies are needed to clarify the pathogenesis mechanisms of the endocrine disorders linked to COVID-19 vaccines.

## 1. Introduction

By 7 January 2024, the COVID-19 pandemic had globally affected more than 774 million people and was responsible for more than 7 million deaths [1]. The emergence of this pandemic led to a global vaccination effort to curb the spread and severity of the disease. Among the most widely approved vaccines are mRNA-based vaccines (BNT162b2 Comirnaty/Pfizer BioNtech; nRNA-1273 Spikevax/Moderna), viral-vector-based (AZD1222 Vaxzevria (ChAdOx1) Oxford University/AstraZeneca; Ad26.COV2.S/Janssen), and inactivated-virus-based (Covilo, BBIBP-CorV(Vero Cells)/Sinopharm) [2]. While SARS-CoV-2 primarily affects the respiratory system, causing pneumonia and acute respiratory distress syndrome in the most severe cases, numerous extrapulmonary manifestations, including endocrine system disorders, can lead to permanent disability in COVID-19 survivors [3]. The structure of SARS-CoV-2 includes the spike (S) protein, which is crucial for the virus’s ability to bind to the host cell receptor, and angiotensin-converting enzyme 2 (ACE2), which facilitates viral entry into host cells [4]. Studies identified multiple epitopes on the SARS-CoV-2 spike protein targeted by T cells and antibodies in COVID-19 patients. These epitopes are crucial for designing vaccines and diagnostic purposes, as they represent the specific parts of the virus recognized by the immune system [5]. In the context of endocrine disorders following COVID-19 vaccination, it is essential to consider how the immune response to these epitopes might influence or interact with endocrine function. For instance, there have been reports of thyroid dysfunction, diabetes, and adrenal insufficiency following vaccination, suggesting a complex interaction between the immune response to the vaccine and endocrine health [6]. The vaccines, which are designed to elicit a robust immune response to specific SARS-CoV-2 epitopes, might also lead to unintended effects on the endocrine system. The mechanisms behind these disorders are similar to those seen in endocrine dysfunction after COVID-19 infection. ACE2 expression in these tissues is a key target for viral damage. Additionally, activating the renin-angiotensin system due to ACE receptor downregulation could also contribute to endocrine dysfunction following COVID-19 vaccination [6,7]. Understanding the targeting epitopes and the immune mechanisms activated by the vaccine can help to assess these risks and manage potential endocrine disorders post-vaccination.

In addition, vaccines might trigger or exacerbate preexisting autoimmune tendencies in susceptible individuals and induce cytokine production, potentially disrupting endocrine function [6,7]. A recent systematic review confirmed the safety of the four most efficacious COVID-19 vaccines [8], even if a few severe adverse events, including endocrine dysfunction, have been reported [9]. This review delves into the current findings on the influence of COVID-19 vaccines on the endocrine system, exploring the specific endocrine disorders reported, the types of vaccines associated with these adverse effects, and the underlying pathogenic mechanisms. We also discuss the criteria that are relevant in establishing causality between adverse events of particular interest related to the endocrine system and vaccine exposure at the population and individual levels and the implications of these findings for clinical practice and future research.

## 2. Materials and Methods

PubMed/MEDLINE, Embase database (Elsevier), and Google Scholar searches were performed for free-text words and medical subject heading terms related to “severe acute respiratory syndrome coronavirus 2 (SARS-CoV-2) vaccine”, “COVID-19 vaccine”, “SARS-CoV-2 vaccination”, “COVID-19 vaccination”, and “SARS-CoV-2 immunization”, variously combined with “endocrinopathies”, endocrine adverse events”, “SARS-CoV-2 vaccine-related”, “endocrine system”, “thyroid”, “subacute thyroiditis”, “Graves’ disease”, “hypothyroidism”, “hyperthyroidism”, “adrenal”, “adrenal insufficiency”, “adrenal crisis”, “Addison disease”, “adrenal hemorrhage”, “pituitary gland”, “hypophysitis”, “hypopituitarism”, “pituitary apoplexy”, “type 1 diabetes”, “diabetes”, “pancreatitis”, “diabetic ketoacidosis”, “ovary”, “amenorrhea”, “menstrual cycle”, “menstrual dysfunction”, “female fertility”, “male infertility”, “couple infertility”, “sperm”, “reproductive health”, “male hypogonadism”, and “sexual dysfunction.” Case reports, case series, original studies, and reviews written in English and published online up to 31 August 2023 were selected and reviewed. The final reference list was defined based on the relevance of each paper to the scope of this review.

## 3. SARS-CoV-2 Vaccination and Thyroid Dysfunction

Following the worldwide rollout of SARS-CoV-2 vaccination initiatives, various immune-mediated inflammatory disorders have been associated with the administration of vaccines [10,11]. Among the reported endocrine side effects, thyroid disorders are the most common [12]. These side effects are presumably triggered by cross-reactivity between the vaccine-targeted virus spike protein and thyroid follicular cell antigens [13]. Another proposed mechanism is an enhancement of autoimmunity by vaccine adjuvants in predisposed individuals in the form of the post-vaccination syndrome known as the autoimmune/inflammatory syndrome induced by adjuvants (ASIA) [13,14]. Current literature describes a spectrum of thyroid dysfunction post-SARS-CoV-2 vaccine administration, including subacute thyroiditis (SAT), silent thyroiditis, overt hypothyroidism, Graves’ disease, and Graves’ orbitopathy, ranging from a new-onset disease to a recurrence of a preexisting condition [15].

Subacute thyroiditis is a self-limited inflammatory disease with a genetic predisposition that may present in individuals carrying specific human leukocyte antigen (HLA) haplotypes [16]. Vaccines against influenza virus, swine flu, hepatitis B, and human papillomavirus have been reported as triggering factors for SAT [15]. HLA-B*35 and HLA-C*04 allele genotypes were suggested as responsible for susceptibility to post-SARS-CoV-2 vaccine SAT development. In a case–control study, those allele rates were significantly higher in 14 patients with post-vaccination SAT compared with 100 healthy controls. In addition, more severe thyrotoxicosis affected relatively more patients with homozygous HLA-B*35 and HLA-C*04 alleles [17].

The first report on post-SARS-CoV-2 vaccine SAT included three cases of female health workers who developed clinical symptoms 4–7 days after the administration of an inactivated SARS-CoV-2 vaccine [13]. These case series were followed by numerous other case reports and series on post-vaccine SAT, as summarized in Table 1. 

From thirty-one reviewed articles, 83 cases were retrieved and are summarized in Table 1 [14,15,18,19,20,21,22,23,24,25,26,27,28,29,30,31,32,33,34,35,36,37,38,39,40,41,42,43,44,45,46,47]. The patients’ median age was 41 years (range 26–82), and 61 (73.5%) were women. The clinical picture of SAT was seen on a median of 10 days (range 1–84) after the last vaccination. All the patients had symptoms like sporadic SAT and were treated accordingly. In 76% of cases, SAT resolved without sequelae, and hypothyroidism had developed in 12% of cases.

Regarding the vaccine type, SAT was reported more frequently in connection with mRNA vaccines, with 51 cases following mRNA vaccines compared with 17 and 15 cases following viral and inactivated vaccines, respectively. Whether a specific vaccine type was associated with a greater risk of SAT was studied in an extensive case/non-case study that surveyed the adverse effects of mRNA vaccines (BNT162b2; mRNA-1273) and viral vector vaccines (ChAdOx1-S; Ad26.COV2.S) in a cohort of 1,221,582 individuals and uncovered 162 cases of subacute thyroiditis. Initially, this study demonstrated that mRNA vaccines were more frequently associated with SAT than viral vaccines. However, this association disappeared when these COVID-19 vaccines were compared with influenza vaccines [48].

Later, two case series compared cohorts of post-vaccine SAT to classical SAT in terms of diagnosis, clinical course, and outcomes. The case series of 23 cases of post-vaccine SAT compared with 62 cases of “classical” SAT showed a longer SAT duration (median 28 days (range 10–150)) in vaccinated patients [49]. Another case series compared the clinical features of SAT between 16 vaccinated and 39 non-vaccinated patients and found no differences in diagnosis and clinical course [50]. Finally, a multicenter retrospective cohort study compared the SAT clinical parameters and outcomes in 258 patients with post-vaccine SAT to patients with classical SAT and showed a similar disease course [51].

Concerning Graves’ disease (GD), 70 cases were retrieved from 26 reviewed case reports and case series (Table 1) [52,53,54,55,56,57,58,59,60,61,62,63,64,65,66,67,68,69,70,71,72,73,74,75,76,77,78]. The median age was 43 years (range 22–74), and 51 (73%) were women. A median latency period between the vaccination date and the diagnosis was 11 days (range 10–40). Ten cases (14%) were patients diagnosed with a relapsed disease.

In a relatively sizeable case series that included 20 patients with a post-vaccination new-onset GD and 44 non-vaccinated patients with the first GD episode, clinical features and follow-up were assessed. The cohort of patients with post-vaccination GD was characterized by a higher male prevalence; older age at the disease onset; and a better response to treatment, both biochemically and immunologically [79].

Another monocentric retrospective study that included 44 patients with post-vaccination GD showed a female prevalence of 43/44 (97.7%), with a mean age of 48.9 (SD 15.6) years. Notably, 7/44 (15.9%) of patients had a history of additional autoimmune disease, and 11/44 (25%) were smokers. The study also suggests the SARS-CoV-2 vaccination impact on GD incidence by comparing the year 2020, when there was a slight increase in GD cases, with the year 2021, when the incidence of GD more than doubled [80].

In contrast, a population-based, matched, case–control study that included 726 patients with GD and 1452 matched controls found no association between SARS-CoV-2 vaccination and GD incidence. While a similar proportion of GD patients and controls received vaccination [80% (581/726) vs. 77.8% (1129/1452), *p* = 0.22], no association was found in univariate analyses between at least one vaccine dose and the disease rates [odds ratio 95% confidence interval: 1.15 (0.92–1.43)] [81]. Furthermore, a recent population-based cohort study of patients with COVID-19 showed an increased risk of GD development compared with the non-COVID-19 group [aHR: 1.30 (95% CI: 1.10–1.54)]. However, after the completion of two vaccine doses, the risk of developing GD, as well as various autoimmune diseases, decreased in the vaccinated versus unvaccinated population [82]. 

Finally, another population-based study evaluated the risk of thyroid dysfunction in 2.3 million participants who received at least one CoronaVac or BNT162b2 vaccine dose. The study demonstrated no evidence of a vaccine-related increase in the incidence of clinical thyroid disease. No increased risk was found in subanalyses specifically for patients with GD for inactivated (CoronaVac) and mRNA (BNT162b2) vaccine types and both vaccine doses within 56 days post-immunization [83].

Ten cases of new-onset or worsening of Graves’ ophthalmopathy (GO) were described [61,65,84]. In all but one case, thyroid function tests were normal, but TSH receptor antibody (TRAb) levels were significantly elevated in eight out of ten patients described. It is noteworthy that no triggering effects other than COVID-19 vaccination with the mRNA vaccine were identified before the significant deterioration of stable thyroid eye disease. GD onset or recurrence concurrently with a new onset of GO following SARS-CoV-2 mRNA vaccination was described [47,48,49,50,51,52,53,54,55,56,57,58,59]. New-onset thyroid eye disease after COVID-19 vaccination was also reported in a patient with GD relapse treated with radioactive iodine nine months before the vaccine administration [85].

**Table 1:** Literature summary of the effects of SARS-CoV-2 vaccines on thyroid function.

Adverse Effects	Study Type (Ref.)	No. of Cases	Age Median (Range)	Sex Female (%)	Vaccine Type			Vaccine Dose			Days from the Last Vaccine Median (Range)	Outcome
					mRNA	Viral	Inactivated	1st	2nd	3rd		
**Post-Vaccination** **Subacute** **Thyroiditis** **Diagnosis and Outcomes**	**Subacute Thyroiditis**											
	**Case reports/small series** [15,16,19,20,21,22,23,24,25,26,27,28,29,30,31,32,33,34,35,36,37,38,39,40,41,42,43,44,45,46,47,48]	**83**	**41 (26–82)**	**61 (73.5)**	51	17	15	40	39	4	10 (1–84)	In 76% of cases, post-vaccination SAT resolved without sequelae. Long-term hypothyroidism developed in 12% of cases.
	Retrospective [49]	23	44 (34–49)	12 (52.2)	18	-	5	5	16	2	45 (7–90)	The clinical course of post-vaccination SAT tended to be longer than the classical SAT.
	Retrospective [50]	16	46.4 ± 9.9	37 (67.3)	6	-	10	10	6	-	6.5 (2–20)	Clinical features of post-vaccination SAT were similar to the classical SAT.
	Retrospective [17]	14	43.1 ± 9.3	12(86)	10	-	6	NA	NA	NA	NA (4–11)	Patients with SARS-CoV-2-vaccine-induced SAT had a higher frequency of HLA-B*35 and HLA-C*04 alleles.
	Retrospective [48]	162	47 (27–86)	120 (74)	130	32	-	59	55	1	10.5 (1–87)	SAT was reported more frequently for post-mRNA vaccines compared with viral vaccines.
	Retrospective [51]	258	42 (36–49)	187 (82.5)	199	-	54	62	175	16	20 (10–40)	Post-vaccination SAT had same clinical course and outcomes as classical one.
**Post-Vaccination Graves’ Disease Diagnosis and Outcomes**	**Graves’ Disease**											
	Case reports/small series [52,53,54,55,56,57,58,59,60,61,62,63,64,65,66,67,68,69,70,71,72,73,74,75,76,77,78]	70	43 (22–74)	51 (73)	50	16	1	38	34	1	11 (1–63)	14% of postvaccination cases were relapsed GD.
	Retrospective [79]	20	51 (37–64)	12	14	6	-	5	13	2	9	Patients with post-vaccination new-onset GD had better initial biochemical and immunologic responses to treatment.
	Retrospective [80]	44	48.9 ± 15.6	43	30	14	-	23	21	-	19.9 + 17.6	Large scale SARS-CoV-2 vaccination may have increased the incidence of GD.
	Retrospective [81]	726	40 (30–53)	541	726	-	-	191	281	254	275.7 ± 144.4	No association between COVID-19 vaccination and the incidence of GD.

## 4. Pituitary Gland and SARS-CoV-2 Vaccine

**Figure 1:**
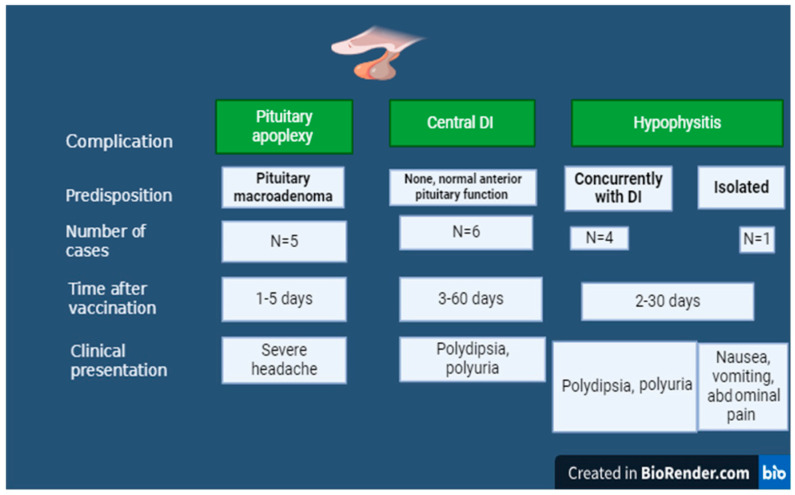
Summary of pituitary adverse events following SARS-CoV-2 Vaccine.

The pituitary gland has been the focus of recent attention due to potential dysfunction following SARS-CoV-2 vaccination. Several cases of pituitary disorders, including hypophysitis, arginine vasopressin deficiency (central diabetes insipidus), inappropriate diuresis, i.e., the syndrome of inappropriate secretion of antidiuretic hormone (SIADH); pituitary apoplexy; and adenocorticotropic hormone (ACTH) deficiency, were described following SARS-CoV-2 vaccination (Figure 1). Most of these patients had no previous known pituitary disease or SARS-CoV-2 infection. The median time between vaccination and the onset of pituitary disorder was three days and ranged from one [86,87,88,89] to sixty days [7]. Descriptions of the reported cases are summarized in Supplementary Table S1.

Regarding pituitary apoplexy (PA), five cases were reported [86,87,88,89,90]. Pituitary apoplexy is a rare but severe condition caused by an abrupt hemorrhaging and/or infarction of the pituitary gland. Its prevalence was estimated at 6·2 cases/100,000 inhabitants [91]. About 50% of PA cases are uncovered by precipitating events like pregnancy, post-partum, coagulopathy, trauma, or hypertension [92]. The most prominent symptom of PA is a severe headache [93], and it was present in four of the five case reports presented in this review [86,87,88,90]. Recently, a potential association of PA with SARS-CoV-2 vaccine was postulated [94]. In four of the five PAs reported following COVID-19 vaccination, a subsequent diagnosis of pituitary adenoma was made [86,88,89,90]. Therapeutic strategies for PA are controversial, i.e., neurosurgery vs. conservative approach [93]. In the cases reported here, only two patients underwent transsphenoidal surgery [86,87,88,89]. Among the five cases of PA reported after COVID-19 vaccination, three occurred after the administration of the viral vector vaccine [87,88,90]; one after the mRNA-based vaccine [86]; and in the last case, the type of vaccine was not reported [89]. The mechanisms of pituitary apoplexy following COVID-19 vaccination are not fully understood, but several hypotheses were suggested. Autoimmunity and vaccine-induced thrombotic thrombocytopenia syndrome (VITT) may be the causes of PA [86]. While autoimmune/autoinflammatory syndrome induced by adjuvants (ASIA) was proposed as a potential factor in the development of PA, an alternative hypothesis suggests that the pituitary gland’s extensive and fragile vascular network may render it particularly vulnerable to PA, especially in the presence of a preexisting pituitary tumor or previous asymptomatic SARS-CoV-2 infection, as evidenced by immunohistochemistry analysis [86,95]. Concerning hypophysitis, this is a sporadic disease with an incidence of ~1 in 9 million/year in the form of primary hypophysitis [96]. Hypophysitis following the SARS-CoV-2 vaccine presents most often as isolated arginine vasopressin (AVP) deficiency (central diabetes insipidus) due to infundibuloneurohypophysitis [7,97,98,99]. AVP deficiency was also reported with the subsequent development of optic neuritis [100]. Rarely, a concomitant anatomic and functional involvement of the anterior pituitary is present [101,102]. Isolated ACTH deficiency [103] and SIADH [104] have also been reported after COVID-19 vaccination. All the cases of hypophysitis presented in Supplementary Table S1 occurred after vaccination with mRNA types of vaccines except one [98]. The pathophysiology of vaccine-induced hypophysitis is not fully understood, but several mechanisms were proposed. ASIA is frequently cited, but three major criteria should be met for its diagnosis. In brief, the major criteria for the diagnosis of ASIA are exposure to external stimuli (infection, vaccine, silicone, adjuvant) before the onset of symptoms, the presence of typical clinical manifestations that include myalgia, myositis, muscle weakness, fever, arthralgia, neurologic manifestations, cognitive impairment, and significant clinical improvement after removal of the provoking agent [105]. In subjects with genetic predisposition, exposure to adjuvants may rarely set off polygenic autoimmune phenomena [106]. Another possible mechanism is that in some circumstances, SARS-CoV-2 mRNA vaccines may induce the production of high and possibly toxic amounts of the spike (S) protein, which may increase the risk of developing adverse reactions [107,108]. Vaccine-induced hypophysitis may affect corticotroph, gonadotroph, and thyreotroph cells. The clinical features vary depending on the extension and severity of the inflammatory process [94].

## 5. Adrenal Glands and SARS-CoV-2 Vaccine

Adrenal glands, with their high blood supply, are susceptible to sepsis-induced damage, endothelial injury, and hemorrhage due to their unique vascular structure. This vulnerability can lead to acute adrenal insufficiency, which is a life-threatening condition. Acute adrenal infarction [109,110] and adrenal hemorrhage [111,112] were described as secondary to SARS-CoV-2 infection. Similarly, adrenal adverse events following COVID-19 vaccination have been reported. These events are rare but may lead to adrenal crisis (AC), which is a life-threatening condition [113,114]. A case series report of five patients with known adrenal insufficiency (AI), three with primary and two with secondary AI, who developed AC within the first 24 h after administration of the first dose of the Astra-Zeneca ChAdOx1 SARS-CoV-2 vaccine was published [115]. The authors concluded that the COVID-19 vaccination could have precipitated AC in these patients. The Clinical Advisory Panel of the Addison’s Disease Self Help Group (ADSHG) has advised that there is no need to routinely increase the glucocorticoid dose in patients with adrenal insufficiency at the time of vaccination if there are no significant symptoms but recommended to increase the maintenance glucocorticoid dosage immediately after experiencing any symptoms following their COVID-19 vaccination [116]. The Pituitary Society surveyed its members to gather insights on the planned management strategies for glucocorticoid administration in patients with known AI after the administration of COVID-19 vaccination. About two-thirds of the responders would increase the glucocorticoid dose only in the case of side effects, like fever, myalgia, or arthralgias [117]. In a study of patients with primary and secondary AI, Pilli et al. found that COVID-19 mRNA vaccines were well tolerated. They did not require increased glucocorticoid dose replacement therapy before vaccination [118]. 

A pheochromocytoma crisis was described in a 63-year-old patient with no medical history one day after administration of the non-replicating viral vector-based vaccine from Johnson & Johnson. A 7 cm adrenal mass was removed, and the patient recovered. The authors concluded that the pheochromocytoma crisis may have been triggered by the COVID-19 vaccine [119]. 

Markovic et al. [120] reported the occurrence of AC in a patient with hypopituitarism after a mRNA-based vaccine (BNT162b2). Data related to non-hemorrhagic adrenal adverse events are summarized in Supplementary Table S2. To our knowledge, seventeen cases of adrenal bleeding following COVID-19 vaccination have been reported to date [121,122,123,124,125,126,127,128,129,130,131,132,133]. Four were unpublished but reported in a systematic review and survey from the UK [133]. All the cases reported occurred after viral vector-based vaccines, except two following the BNT162b2 SARS-CoV-2 mRNA vaccine [133]. The main findings of these studies are summarized in Supplementary Table S3. The pathophysiological mechanisms behind adrenal adverse events following COVID-19 vaccination are not fully elucidated, but several hypotheses and observations have been made. Adrenal crisis (AC) can be precipitated by the stress response to vaccination, particularly in individuals with preexisting adrenal insufficiency or hypopituitarism [120]. Adrenal hemorrhage, which is a severe complication, has been reported in the context of vaccine-induced immune thrombotic thrombocytopenia (VITT). VITT, which is characterized by venous thrombosis, is a rare but serious adverse event associated with adenoviral vector-based COVID-19 vaccines and carries a high mortality risk [134]. The pathogenesis of VITT involves the production of antiplatelet factor 4 (PF4) antibodies triggered by the vaccines, which can lead to platelet activation, thrombosis, and thrombocytopenia [135]. Adenoviral vector-based vaccines have been more frequently implicated in VITT cases, while mRNA-based COVID-19 vaccines have also been linked to instances of adrenal insufficiency. It is suggested that the vaccine’s components, such as adjuvants, might trigger an immune response that could potentially lead to endocrine dysfunctions, including adrenal insufficiency [103].

## 6. SARS-CoV-2 Vaccination and Female Reproductive System

Generally, vaccines do not harm fertility [136]. Still, the SARS-CoV-2 vaccine-related immune alterations linked to potential infertility have raised public concern. The absence of safety evidence due to excluding pregnant women from vaccine trials may have triggered initial apprehension [137]. Moreover, as was previously claimed and later criticized, due to the resemblance of the SARS-CoV-2 spike protein and syncyntin1, the mRNA vaccine could theoretically harm placental function, raising the risk of miscarriage by stimulation antibodies against the syncyntin1 protein [138]. Finally, a study indicated that T-cell activation increased the risk of implantation failure after the transfer of in vitro fertilized embryos [139].

In light of these concerns, clinical studies examined the risk of infertility by investigating the SARS-CoV-2 vaccine-related effects on the menstrual cycle and ovarian reserve. Ovarian reserve markers include antral follicle count (AFC), anti-Müllerian hormone (AMH), follicle-stimulating hormone (FSH), and estradiol (E2). These markers may reflect oocytes’ quantity, quality, and reproductive capacity, whereas alterations in female sex hormone levels may cause menstrual cycle irregularities [140]. 

During the COVID-19 pandemic, more patients have experienced menstrual cycle alteration after SARS-CoV-2 vaccination. A retrospective study in young women (mean age 33 years) reported delayed menstruation and abnormal uterine bleeding in 23% and 77% of cases, respectively. Those abnormalities occurred within the first three weeks of vaccination, mainly after the second vaccine dose. It appears that the mRNA vaccines (Pfizer/BioNTech Comirnaty and Moderna) were most commonly associated with reports of menstrual dysfunction related to the alteration of female sex hormone levels. However, FSH and E2 levels were not significantly different in the vaccinated women compared with the control group and did not correlate with the vaccine types [141]. Another study reported no alterations in hormone levels, including E2, FSH, and LH, after the third dose of the SARS-CoV-2 vaccine compared with pre-vaccination levels [142].

The association between SARS-CoV-2 vaccination and ovarian reserve parameters was investigated in twelve studies summarized in Table 2 [143,144,145,146,147,148,149,150,151,152,153,154,155].

Four studies [143,144,145,146] showed no significant shift in AMH and AFC levels following vaccination. However, one prospective study described a significant decrease in serum AMH levels at months 3 or 6 of follow-up compared with pre-vaccination. However, at month 9 of follow-up, serum AMH levels normalized and stayed within the normal ovarian reserve range (>1.1 ng/dL) throughout the study period [147]. 

Eight reports [141,142,143,148,149,150,151,152] focused on the population undergoing assisted reproduction treatment and demonstrated no harmful impact of the SARS-CoV-2 vaccine on ovarian reserves during assisted reproduction cycles. One of the prospective studies showed that regardless of the type of vaccine, there was no significant adverse effect on ovarian function in patients treated with assisted reproduction therapy [155]. 

Finally, a recent prospective Internet-based study enrolled couples who were trying to conceive without the use of fertility treatment. The study found no link between the COVID-19 vaccine and an increased risk of miscarriage and no evidence of a higher risk of miscarriage associated with male partner vaccination [168].

## 7. SARS-CoV-2 Vaccination and Male Reproductive System

Hypothetically, a direct impact of the SARS-CoV-2 vaccine on male fertility through compromised germ cell development would be reflected in a prompt drop in semen quality [169]. As is known, testicular spermatozoa form and mature within 2.5 months and follow the final differentiation into epididymal sperm during the next two weeks. One should consider the spermatogenic cycle length when assessing the interval between vaccination and semen analysis. Regarding the potential effect of the vaccine on semen quality, it would be appropriate to sample the specimen within three to six months after vaccination. Semen quality parameters include the pH, volume, morphology, total sperm count, sperm concentration, progressive motility, total progressive motile sperm count, and chromatin condensation [170].

Twelve clinical studies evaluated the SARS-CoV-2 vaccine’s effect on male fertility by analyzing the semen quality, as summarized in Table 2. Four prospective studies in healthy young men evaluated sperm parameters, and no significant abnormalities were detected [156,157,158,159]. However, a study on healthy men undergoing IVF due to female infertility showed a substantial reduction in total and progressive sperm motility after vaccination, which remained in the normal range [160]. Six other retrospective studies examined various vaccine types (mRNA, inactivated, and viral vector vaccines) on sperm quality characteristics. Three studies investigated healthy men [161,162,163] and three individuals undergoing fertility treatments [164,165,166]. Overall, sperm characteristics were not influenced after vaccination, excluding a temporary decrease in concentration and motility in one study and decreased total sperm motility with increased FSH levels compared with the men in the unvaccinated group in another study; both studies focused on healthy donors [164,166]. In addition, in the survey of men undergoing fertility treatments, there was an insignificant drop in sperm volume [165]. 

Finally, a recent study demonstrated the presence of SARS-CoV-2 antibodies in seminal plasma and assessed its impact on sperm quality. The study showed a strong correlation between titers of SARS-CoV-2 antibodies in serum and seminal plasma. However, considerable levels of the antibodies in seminal plasma after vaccination were not associated with a decline in sperm quality [166].

## 8. Diabetes Mellitus and COVID-19 Vaccine

### 8.1. Diabetes Mellitus and SARS-CoV-2 Vaccination

**Figure 2:**
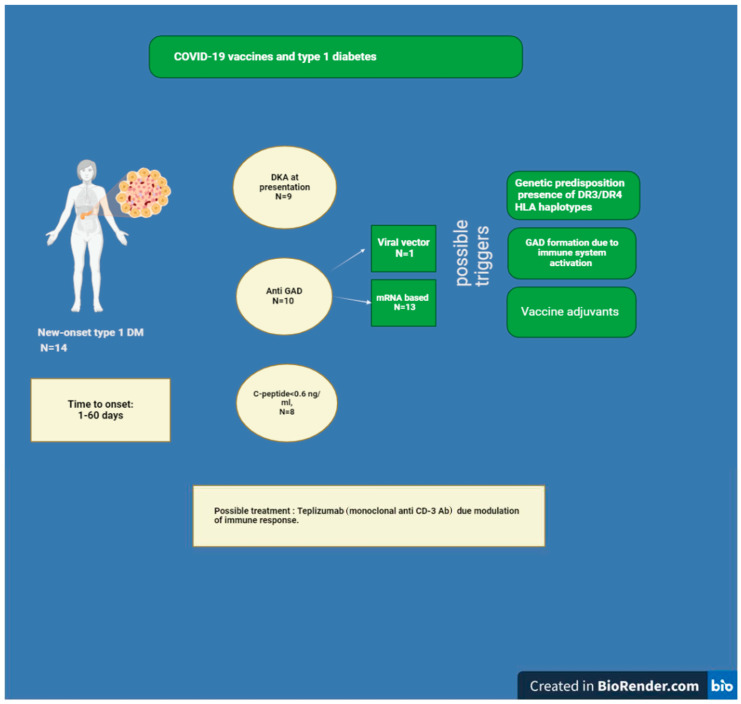
Type 1 diabetes following SARS-CoV-2 Vaccination.

Diabetes is a risk factor for mortality regarding the SARS coronavirus, the MERS coronavirus, influenza A 2009 (H1N1), and SARS-CoV-2 infections. Increased airway glucose levels can raise the replication of respiratory pathogens, exposing diabetic patients to bacterial overgrowth after a viral infection [171]. Safe and effective messenger RNA (mRNA) vaccines successfully aborted the severe acute respiratory syndrome coronavirus 2 (SARS-CoV-2) pandemic [172]. Nevertheless, type 2 diabetes patients with poor glycemic control after receiving the mRNA-BNT162b vaccine had an increased incidence of SARS-CoV-2 breakthrough infections [173]. How mRNA vaccines impact islet cells and the central mechanism of reducing insulin secretion after COVID-19 vaccination remains unresolved. A few cases of pancreatitis related to SARS vaccination were reported [174,175,176]. Despite increasing evidence suggesting that COVID-19 vaccination may be associated with new-onset autoimmune diseases, the causal relationship between COVID-19 vaccines and these autoimmune conditions, as well as the underlying mechanisms, particularly immune cross-reactions, remains to be conclusively demonstrated [177]. While SARS-CoV-2 vaccination is generally advised for individuals with diabetes, and the majority of those with preexisting type 1 or type 2 diabetes have been vaccinated without significant issues, there are potential short-term effects on glucose control that can manifest as diabetic ketoacidosis in both diabetic and non-diabetic individuals [178,179,180,181,182]. A comprehensive systematic review examined the relationship between COVID-19 vaccination and diabetes. This review included 54 studies and found that individuals with diabetes, particularly those with poor glycemic control, may experience an increased risk of blood glucose elevation following vaccination compared with the general population. Additionally, the immune response to the vaccine tends to be lower in diabetic patients than in non-diabetic individuals [178]. The number of reported cases of new-onset type 1 diabetes following mRNA COVID-19 vaccination varies across different studies and reports. According to a recent systematic review, among 12 patients diagnosed with type 1 diabetes following COVID-19 vaccination, 10 cases were linked to mRNA vaccines, with a significant proportion of these diagnoses occurring after the administration of the second dose [180,183]. Another case of a 39-year-old woman who developed fulminant type 1 diabetes after mSARS-CoV-2 vaccination was reported [184]. In addition, several cases of new-onset type 1 diabetes presenting as diabetic ketoacidosis (DKA) were reported [182,185,186]. Notably, the unusual progression of prediabetes to type 1 diabetes with ketoacidosis [187] and the conversion of preexisting type 2 into type 1 autoimmune diabetes concomitant to the occurrence of Graves’ disease was also described [58]. However, based on the information provided in the literature reviewed in our study, we cannot confidently state an incidence rate or calculate a pooled rate for new-onset diabetes following COVID-19 vaccination. More research specifically focusing on this topic is needed to provide a definitive incidence rate. The time of onset of type 1 diabetes after vaccination is variable and ranges from 1–3 days [182,186,188] to 2–8 weeks after vaccination [180,181,185,187,189]. Low C-peptide levels were found at diagnosis in all the cases mentioned above. Only in one of the patients reported by Aydoğan Bİ was the C peptide level at the lower end of the normal range [181].The data about type 1 diabetes following SARS-CoV-2 Vaccination is summarized in Figure 2.

Regarding immunologic testing, the presence of autoantibodies was not uniform. The insulinoma-associated antigen-2 (IA-2) antibody was negative in all studies except two patients [179]. The anti-glutamic acid decarboxylase (GAD) antibody was not detected in some studies [182,185,186], while high titers of autoantibodies were detected in others [179,180,181,189]. High insulin autoantibody (IAA) levels were also reported [190].

Pancreatic damage following COVID-19 vaccination involves specific activation of the innate immune system, marked by the overexpression of cytoplasmic retinoic acid-inducible gene I (RIG-I)-like receptors and melanoma differentiation-associated gene 5 (MDA5) in both β- and α-pancreatic cells [190]. The MDA5 gene is implicated in the immune response against SARS-CoV-2 infection and may also enhance immune activation following mRNA vaccination [191]. Although most cases of type 1 diabetes were reported following mRNA vaccines, some were also reported after whole inactivated virus-based COVID-19 (COXAVIN) and non-replicating viral vector COVISHIELD (ChAdOx1 nCoV-19) vaccines [178].

### 8.2. Could the COVID-19 Vaccine Elicit GAD Antibody Formation?

GAD is the enzyme related to gamma-aminobutyric acid (GABA) production, which is a central brain inhibitory neurotransmitter. High levels of GABA were found in pancreatic β-cells and shown to play a role in the activation of insulin release and inhibition of β-cells apoptosis [192]. GAD antibodies were found in several neurologic syndromes, including stiff-person syndrome, cerebral ataxia, epilepsy, and limbic encephalitis [193]. The central mechanism in GAD antibody formation lies in activating a specific T helper cell population related to the synthesis of proinflammatory cytokines [194]. GAD antibodies are more common than insulinoma-antigen 2 (IA-2) antibodies in older patients at the time of type 1 diabetes diagnosis. A case of anti-GAD positive limbic encephalitis developed 20 days after administration of the second dose of the COVID-19 mRNA (BNT162b2) vaccine was reported [195]. Since vaccination itself can lead to increased levels of the inflammatory response [196], it can subsequently trigger anti-GAD formation and probably be involved in developing type 1 diabetes or neurologic complications related to anti-GAD antibodies.

### 8.3. Relationship between Immune Checkpoint Inhibitors Therapy and the Onset of Type 1 Diabetes Following SARS-CoV-2 Vaccination

Checkpoint inhibitors represent a novel category of cancer treatments that have been utilized over the past ten years, with a focus on blocking the cytotoxic T-lymphocyte antigen 4 (CTLA-4) and programmed cell death protein 1 (PD-1) pathways. While these treatments showed substantial therapeutic benefits, they were also associated with severe complications, such as the sudden onset of type 1 diabetes due to the destruction of β-cells [197]. A recent comprehensive analysis determined that administering COVID-19 vaccines to cancer patients undergoing treatment with immune checkpoint inhibitors is considered safe despite initial concerns regarding potential immune-related side effects [198]. Nevertheless, several cases of type 1 diabetes triggered by mRNA-based SARS-CoV-2 vaccines in patients given nivolumab [188,199] or pembrolizumab [200] were reported. 

### 8.4. Adjuvants and Type 1 Diabetes

In 2011, Shoenfeld aimed to classify a number of autoimmune, autoinflammatory, and other autonomic phenomena under a new nosology unit named ASIA. The originality of this entity lies in one unifying feature: the administration of different adjuvants [201].

Adjuvants are numerous substances added to vaccines or medical devices for the accentuation of immune responses [198]. Among them, aluminum salts were commonly included in different vaccines [202]. When adjuvants predominantly activate the adaptive immune system, they trigger manifestations of autoimmune conditions, whereas activation of the innate immune system leads to autoinflammatory diseases [203]. Among 500 cases of autoimmune and autoinflammatory diseases potentially linked to the hepatitis B and papillomavirus vaccines, 13 were associated with a new onset of type 1 diabetes [202]. Compelling evidence that type 1 diabetes develops because of vaccine adjuvants is missing.

Autoimmune syndromes, including Guillain–Barre syndrome, vaccine-induced immune thrombotic thrombocytopenia (VITT), immune thrombocytopenic purpura, autoimmune liver disease, Ig-A nephropathy, and arthritis were described after COVID-19 vaccination [204]. The significant differences between the adjuvants included in COVID-19 vaccines have made it challenging to draw conclusive links between the development of autoinflammatory or autoimmune conditions and the vaccines themselves or their adjuvants due to their complexity [201,205].

## 9. Discussion

Vaccines represent one of the most essential and powerful implements to prevent morbidity and mortality associated with infectious diseases in healthy populations, patients with risk factors, and immunocompromised people [206]. Despite the benefits of vaccination, which outweigh the risk of severe SARS-CoV-2 infection complications, misinformation about adverse events, particularly in relation to the endocrine system, may compromise adherence to public health guidelines [207]. 

### 9.1. Comparative Studies

Several comparative studies were conducted to assess the incidence of health problems after COVID-19 vaccination versus non-vaccinated individuals and evaluate the safety and effectiveness of COVID-19 vaccines. Vaccinated individuals are less likely to experience severe COVID-19, require intensive care, or die from the disease compared with non-vaccinated individuals. This was demonstrated across different populations and with different SARS-CoV-2 variants, including Delta and Omicron. Vaccination also reduces the duration of hospitalization and the severity of symptoms in those who do contract COVID-19 [208,209]. 

Vaccinated individuals also have lower overall mortality rates compared with the non-vaccinated [210]. 

Studies showed that vaccination reduces the incidence and duration of long COVID symptoms [211].

Some key findings regarding the incidence of endocrine adverse events in vaccinated versus non-vaccinated individuals are available from the literature:

A large cohort study showed that COVID-19 vaccination is associated with a decreased risk of orchitis and epididymitis compared with unvaccinated men [212].

A study analyzed electronic medical records to assess the association between COVID-19 and autoimmune diseases, including Graves’ disease. The findings showed that COVID-19 vaccination attenuated the risk of COVID-19-induced autoimmune diseases, including Graves’ disease, suggesting a protective effect rather than an increased risk of autoimmune diseases [82].

In a large-scale, controlled, population-based study, no association was found between COVID-19 vaccination and the incidence of Graves’ disease [81]. 

Another recent population-based controlled retrospective study found no correlation between COVID-19 infection or vaccination and subacute thyroiditis [213].

In summary, available evidence indicates that the incidence of endocrine adverse events post-COVID-19 vaccination is generally low and comparable with that in non-vaccinated individuals. These findings support the safety of COVID-19 vaccines concerning endocrine health, noting that rare adverse events that are typically manageable.

### 9.2. Causality

A causality assessment of an adverse event following immunization (AEFI) is challenging. The causality of an AEFI should be determined at the population and individual levels [214]. 

Six criteria are relevant to establish causality at the population level:

- Temporal relationship: vaccine exposure must precede the event, which is the only criterion essential to establish causality.
- Strength of association, which is based on the statistical analysis of the extensive AEFI database, like the Vaccine Adverse Event Reporting System (VAERS) in the USA [215], or other country-specific systems, like the COVID-19 Vaccine Safety Research Center established in September 2022 at the request of the Korea Disease Control and Prevention Agency [216].
- Dose–response relationship, but in the case of vaccines, these parameters are generally fixed.
- Consistency of evidence.
- Specificity: the vaccine is the only trigger of the adverse event.
- Biological plausibility and coherence: the association between the AEFI and the vaccine should be compatible with the current knowledge of the biology of the vaccine and the AEFI.

The United States Institute of Medicine has applied these criteria to the revised WHO causality algorithm [217]. 

At the individual level, it is usually not possible to determine with certainty a causal relationship between a specific AEFI and a particular vaccine. The information obtained from the population-based data will undoubtedly influence the assessment of causality at the individual level. 

Recent studies and accumulating reports highlighted the potential endocrine adverse events associated with SARS-CoV-2 vaccines. Based on the search results provided, we aim to provide a synthesized and balanced understanding of the relationship between COVID-19 vaccines and endocrine disorders.

Several endocrine organs were identified to be affected by the SARS-CoV-2 vaccine. 

### 9.3. Thyroid Disorders

Among the post-vaccination new-onset endocrine disorders, subacute thyroiditis (SAT) has been the most reported in the literature, with several hundred published cases. Garcia M et al. [48] analyzed the potential association between subacute thyroiditis (SAT) and COVID-19 vaccines using the EudraVigilance database. During the study period, a total of 627,500,000 doses of COVID-19 vaccines were administered in the European Union/European Economic Area. Out of these doses, 1,221,582 cases of adverse reactions were registered in EudraVigilance for these vaccines. Among the adverse reactions, there were 174 cases of subacute thyroiditis: 110 with BNT162b2, 30 with mRNA-1273, 32 with ChAdOx1-S, and 2 with Ad26.COV2.S. The reporting rate of subacute thyroiditis was 0.3 cases per 1 million doses administered. The majority of cases (64.8%) were classified as severe according to the EU criteria, with most of these being medically important conditions (68.6%). There was some disproportionate reporting for subacute thyroiditis with BNT162b2, mRNA-1273, and ChAdOx1-S vaccines compared with other drugs. The reporting odds ratio (ROR) for subacute thyroiditis was the highest for BNT162b2 (ROR = 5.30, 95% CI 4.23–6.65), followed by mRNA-1273 (ROR = 4.21, 95% CI 2.84–6.22) and ChAdOx1-S (ROR = 1.88, 95% CI 1.30–2.71). Most reviewed studies demonstrated similarities between post-vaccine SAT and the classic disease regarding diagnosis, clinical course, and outcomes [48,49]. The underlying pathophysiology is an inflammatory process that causes the destruction of follicular epithelium and may be triggered by an immune response following vaccination. However, data establishing a direct causative relationship are lacking. Graves’ disease (GD) was also reported in connection with the vaccination in 70 case reports, small series, and three clinical studies, including a large population-based, matched case–control study of 4.7 million people that found no association between GD and the COVID-19 vaccine [52,53,54,55,56,57,58,59,60,61,62,63,64,65,66,67,68,69,70,71,72,73,74,75,76,77,78,79,80,81,82,83]. According to the raw data from the VAERS system, 391 cases of autoimmune thyroiditis and 12 reports of Graves’ disease were reported after COVID-19 vaccination, but underreporting is probable. In contrast, only one case of Graves’ disease and no cases of autoimmune thyroiditis were reported after the influenza vaccine.

Nevertheless, two retrospective studies did not find an increase in the incidence of Graves’ disease after COVID-19 vaccination. In the study of Endo M et al., there was no increase in the incidence of Graves’ disease after the implementation of COVID-19 vaccination [218]. In the large population-based study of Gorstein A. et al., no association between COVID-19 vaccination and the incidence of Graves’ disease was found [81]. Interestingly, SARS-CoV-2 itself was found to be associated with autoimmunity, and COVID-19 vaccination could be related to its protective effects [82]. A meta-analysis of 21 publications comprising 57 post-vaccination GD cases found that the majority of the cases reported were new-onset Graves’ disease. The specific vaccine brands associated with these cases varied, with COMIRNATY being the most commonly reported. Given the rarity of this adverse event, so far, it is not possible to establish a direct causal link between COVID-19 vaccination and Graves’ disease, and more research and monitoring are needed to better understand the potential link between COVID-19 vaccination and Graves’ disease [219].

### 9.4. Diabetes Mellitus

Type 1 diabetes mellitus (T1DM) following vaccination was also observed. The variety of clinical presentations included severe DKA in previously healthy individuals and conversion to T1DM in patients with prediabetes or T2DM conditions [58,180,181,182,183,184,185,186,187,188,189,190]. Pre-diabetic individuals were more likely to develop T1DM following vaccine administration. However, whether insulin resistance plays a part in post-vaccination DKA development is unclear. According to the raw data from the VAERS system, 319 cases of DKA were reported by healthcare personnel and individuals after COVID-19 vaccination.

In comparison, only seven cases of DKA were described after vaccination with the Fluzone Quadrivalent vaccine used for the prevention of influenza disease between 2014 and 2019 and none between 2019 and 2023. The onset of the summarized cases ranged from 1 day to 8 weeks post-vaccination, and the T1DM autoantibody positivity profile was variable. Given the existing evidence of deteriorated glycemic parameters following immunization in patients with diabetes, it still should be elucidated whether the vaccine is responsible for new-onset T1DM or the post-vaccination immune response uncovers preexisting conditions in susceptible individuals. This area requires further investigation. 

### 9.5. Fertility

Evaluating the impact of COVID-19 vaccines on fertility is challenging and was the subject of significant investigation. In most clinical studies, ovarian reserve markers and semen quality analysis were used as the primary assessment tools in female and male fertility, respectively. The summarized studies that evaluated the potential effect of the vaccines on female fertility showed no significant detrimental effects on ovarian reserve markers [143,144,145,146,147,148,149,150,151,152,153,154,155]. Similarly, studies on male fertility showed no adverse effects of the vaccines on the parameters of semen specimens [156,157,158,159,160,161,162,163,164,165,166].

A systematic review and meta-analysis found no significant differences in sperm motility, concentration, or pregnancy rates between vaccinated and non-vaccinated individuals, supporting the safety of vaccines regarding fertility [220]. Due to the limited data available and the variability in study designs and populations, the exact incidence rates of adverse events on the reproductive system following COVID-19 vaccination are not established. 

### 9.6. Rare Adverse Events and Underreporting

Pituitary disorders were initially reported in the setting of SARS-CoV-2 infection, followed by published clinical cases of hypophysitis, central diabetes insipidus, SIADH, pituitary apoplexy, and ACTH deficiency as rare adverse effects of the vaccination. The rarity of these reports, combined with the lack of large-scale studies specifically focused on pituitary adverse events, indicates that the incidence is likely to be very low [221]. The summarized cases of pituitary conditions are not very numerous, but perhaps the actual incidence is underreported, and thus, underestimated [7,86,87,88,89,90,91,92,93,94,95,96,97,98,99,100,101,102,103,104]. In addition, a delay in diagnosis can arise from overlapping symptoms related to pituitary hormone deficiencies and those experienced after vaccination. Therefore, reports on adverse events, particularly those related to rare disorders, such as pituitary diseases, are essential to raise awareness and reporting, and thus, improve outcomes. 

Adrenal insufficiency and other adrenal-related adverse events are rare. The available data are primarily based on case reports and small cohort studies.

Hemorrhagic and non-hemorrhagic adrenal post-vaccination adverse events are also rare phenomena but could be life threatening. Cases of the adrenal crisis were described in this review, both in patients with existing adrenal insufficiency (AI) and in those after adrenal hemorrhage [115,116,117,118,119,120,121,122,123,124,125,126,127,128,129,130,131,132]. For patients with known AI, the literature concerning post-vaccination glucocorticoid dose suggests increasing the maintenance dose in the case of vaccine-related symptoms [116].

### 9.7. Limitations of the Reviewed Studies

We are aware of the importance of critically evaluating the limitations of the studies included in our review. 

The main limitations are as follows:

1. Sample Size and Power

Many of the studies we reviewed, particularly case reports and small case series, had limited sample sizes. This reduces their statistical power and the generalizability of their findings. For example, the case report by Ankireddypalli AR et al. [102] on hypophysitis following COVID-19 vaccination, while informative, cannot establish causality or estimate incidence rates.

2. Selection and Reporting Bias

There is a potential for selection and reporting bias in the literature, with a tendency to publish and report positive findings. This may lead to an overestimation of the association between COVID-19 vaccination and endocrine adverse events. We noted this limitation in interpreting studies, such as the systematic review by Triantafyllidis et al. [219].

3. Lack of Control Groups

Many studies, mainly case reports and series, lack appropriate control groups. This lack makes it challenging to determine whether the observed endocrine events are causally related to vaccination or merely coincidental. The population-based study by Wong CK et al. [83] addressed this limitation to some extent, but such comprehensive studies are rare in the current literature.

4. Heterogeneity in Study Designs and Definitions

The studies we reviewed employed various designs and definitions of endocrine adverse events, making direct comparisons and meta-analyses challenging. This heterogeneity limits our ability to draw definitive conclusions about the overall risk of endocrine disorders following COVID-19 vaccination.

5. Temporal Association vs. Causality

While many studies report a temporal association between vaccination and endocrine events, establishing causality remains challenging. The study by Gorshtein et al. [81] attempted to address this through a matched case–control design, but such rigorous approaches are not universal in the literature.

6. Limited Long-Term Follow-Up

Most studies had relatively short follow-up periods, limiting our understanding of the potential long-term endocrine effects of COVID-19 vaccination. This short follow-up is particularly relevant for conditions that may have a delayed onset or prolonged course.

7. Confounding Factors

Many studies did not adequately control for potential confounding factors, such as age, sex, preexisting conditions, and concurrent medications. The study by Şendur et al. [17] on HLA genotypes and vaccine-induced thyroiditis highlights the complexity of these interactions, but such comprehensive analyses are not common.

8. Variability in Vaccine Types

The studies covered various COVID-19 vaccine types, making it difficult to draw conclusions about specific vaccines. The risk profiles may differ between mRNA, viral vector, and other vaccine types, as suggested by Wong CK et al. [83], but more comparative studies are needed.

9. Potential for Nocebo Effect

The heightened awareness and anxiety surrounding COVID-19 vaccination may contribute to a nocebo effect, potentially inflating the reported incidence of adverse events. This psychological factor is challenging to control for in observational studies.

10. Limited Data on Certain Endocrine Disorders

While some endocrine conditions (e.g., thyroid disorders) are well-represented in the literature, data on others (e.g., adrenal or pituitary disorders) are more limited, creating an imbalance in our understanding of the overall endocrine impact of COVID-19 vaccination.

## 10. Conclusions

The literature reviewed does not provide definitive evidence of a direct causal relationship between COVID-19 vaccination and endocrine adverse effects. The occurrence of endocrine disorders following a population-wide immunization program may reflect causality bias rather than a direct effect of the vaccines. Further research is needed to evaluate the association between endocrine conditions and COVID-19 vaccines more thoroughly.

Despite the reported cases, the data do not challenge the safety and efficacy of the available COVID-19 vaccines. The benefits of vaccination in preventing severe COVID-19 outcomes continue to outweigh the potential risks of endocrine adverse events. Physicians should remain vigilant and report adverse events to contribute to a better understanding of the vaccine’s safety profile and to guide patient care effectively.

Considering these findings, it is recommended that physicians explore the following actions:

- Continue to advocate for COVID-19 vaccination, given the overall benefit in preventing severe disease. Despite a notable reduction in severity and mortality rates in various regions, COVID-19 remains a significant global health concern with continued new cases and societal impacts
- Monitoring patients with preexisting endocrine disorders closely post-vaccination for any changes in their condition.
- Report any suspected adverse events post-vaccination to relevant systems to contribute to safety monitoring and research.
- Consider individual patient risk factors when advising on vaccination and managing post-vaccination symptoms.
- Educate patients about possible adverse events while emphasizing the overall benefits of vaccination.

## Figures and Tables

**Table 2:** Literature summary on the effect of SARS-CoV-2 vaccines on the reproductive system.

Adverse Ef-fects/Primary Study Point	Study Type (Ref.)	No. of Cases	Vaccine Type			Vaccine Dose			Days from the Last Vaccine	Age Mean ± SD	Outcome
			mRNA	Viral	Inactivated	1st	2nd	3rd			
**Female Reproductive System**											
**Ovarian Reserve Markers**	**Retrospective** **[143]**	46	-	-	46	-	46	-	30	36.4 ± 4.9	No effect on AMH level or antral follicle number.
	Retrospective [144]	309	-	-	309	78	257	-	<90 (28%) ≥90 (72%)	31.2 ± 3.8	No effect on pregnancy rate in assisted reproduction therapy.
	Retrospective [145]	146	-	-	146	-	146	-	72.4 ± 57.0	33.7 ± 5.6	No effect on pregnancy rate in assisted reproduction therapy.
	Prospective [146]	129	129	-	-	-	129	-	90	29 ± 5.23	No effect on AMH levels.
	Prospective [148]	74	74	-	-	-	74	-	180	27.6 ± 5.3	No effect on AMH levels.
	Prospective [149]	62	62	-	-	-	62	-	90	26.3 ± 3.6	No effect on FSH, LH, E2, AMH, ovarian volume, or number of antral follicles.
	Retrospective [150]	474	-	-	474	-	474	-	508.0 ± 250.2 (mean ± SD)	30.8 ± 4.95	No effect on AMH levels.
	Prospective [151]	31	31	-	-	-	31	-	180 (median)	35.5 ± 4.7	No effect on AMH levels.
	Prospective [152]	37	37	-	-	-	37	-	14–60	33.3 ± 6.1	No effect on ovarian reserve or pregnancy rate in assisted reproduction therapy.
	Retrospective [153]	36	36	-	-	-	36	-	7–85	37.3 ± 4.6	No effect on ovarian reserve in assisted reproduction therapy.
	Retrospective [154]	142	135	7	-	15	127	-	93 ± 65 (mean ± SD)	34 ± 4	No effect on pregnancy rate in assisted reproduction therapy.
	Retrospective [155]	510	441	69	-	-	510	-	60	34.8 ± 7.7	No effect on pregnancy rate in assisted reproduction therapy.
**Male Reproductive System**											
**Semen Quality Parameters**	Prospective [156]	45	45	-	-	-	45	-	75 (median)	28 (median)	No deleterious effect on sperm quality.
	Prospective [157]	33	33	-	-	-	33	-	≥72	27 (median)	No deleterious effect on sperm quality.
	Prospective [158]	75	75	-	-	-	75	-	37 (mean)	38.6	No deleterious effect on sperm quality.
	Prospective [159]	47	47	-	-	-	47	-	30	29.3	No deleterious effect on sperm quality.
	Prospective [160]	60	60	-	-	-	60	-	≥90	36 (median)	No deleterious effect on sperm quality.
	Retrospective [161]	37	37	-	-	-	37	-	>145	26.1	No deleterious effect on sperm quality.
	Retrospective [162]	43	-	-	43	-	43	-	30.1 (mean)	28.6	No deleterious effect on sperm quality.
	Retrospective [163]	351	-	-	351	8	183	160	112.7 (mean)	35	No deleterious effect on sperm quality.
	Retrospective [164]	106	93	11	-	22	82	-	59 (median)	39 (median)	No deleterious effect on sperm quality and fertilization capacity of men undergoing ART treatments.
	Retrospective [165]	72	72	-	--	-	72	-	71 (mean)	35.7	No deleterious effect on sperm quality.
	Retrospective [166]	105	-	-	105	-	105	-	80.6 (mean)	33.9	No significant differences were observed in sperm quality and IVF outcomes.
	Observational case–control [167]	43	NA	NA	NA	1	17	22	NA	36	No association between SARS-CoV-2 vaccination parameters and markers of sperm quality.

## Supplementary Materials

The following supporting information can be downloaded from https://www.mdpi.com/article/10.3390/vaccines12070750/s1, Table S1: Main characteristics of the case report studies on pituitary adverse events following COVID-19 vaccines included in this review; Table S2: Summarized data regarding adrenal adverse events (other than hemorrhage/infarction) after COVID-19 vaccine; Table S3: Summarized data from case reports regarding COVID-19 vaccine related adrenal hemorrhage (AH) or infarction (AI). References [7,86,87,88,89,90,97,98,99,100,101,102,103,104,115,119,120,121,122,123,124,125,126,127,128,129,130,131,132] are cited in the Supplementary Materials.
