# Supplementary material for: SARS-CoV-2-Vaccine-Related Endocrine Disorders: An Updated Narrative Review
Source: Vaccines (Basel). 2024 Jul 8;12(7):750. doi: 10.3390/vaccines12070750 (PMC11281608; doi:10.3390/vaccines12070750)
Supplement: Supplementary file 1 [file vaccines-12-00750-s001.zip › Table S1.pdf]

Supplementary Table S1: Main characteristics of the case-report studies on pituitary adverse events following COVID-19 vaccines included in this review.

Adverse effect	Author (Ref)	Vaccine type	Latency between vaccine (dose) and symptoms (days)	Sex (M/F)	Age (years)	Clinical Presentation	Treatment	Outcome
Pituitary apoplexy	Aliberti et al [86]	Moderna mRNA 1273-	1 day after 3d dose	M	50	Persistent headache, nausea, vomiting, fever, diplopia MRI disclose a recent hemorrhage in a large pituitary macroadenoma Secondary hypogonadism	Stress doses of dexamethasone Transsphenoidal resection of the pituitary tumor	Recovery without hormonal deficits
Pituitary apoplexy	Roncati et al [87]	viral vector based Vaxzevria ChAdOx1	1st day after the 1st dose	F	28	Worsening tension headache after the 2d dose Amenorrhea, Hyperprolactinemia MRI showed an hemorrhagic lesion in the pituitary	Not reported	Recovery without surgery
Pituitary apoplexy	Zainordin et al [88]	Viral Victor based Astra Zenica ChAdOx1	1st day after 2nd dose	F	24	Severe headache without other symptoms MRI suggestive of bleeding in preexisting pituitary adenoma with mass effect on chiasma Normal anterior pituitary function except elevated IGF1 levels with suppressed GH levels after oral glucose tolerance test	High dose steroid therapy	Improvement in symptoms 50% reduction in pituitary mass without surgery
Pituitary apoplexy	Piñar-Gutiérrez et al [90]	viral vector based Vaxzevria ChAdOx1	5 days after the 1st dose	F	37	Severe frontal headache. At MRI, bleeding in a10 mm pituitary adenoma without chiasma involvement. No hormonal deficits	None	Recovery within 2-3 weeks
Pituitary apoplexy	Jaggi S et al [89]	Not reported	1 day after 2nd dose	M	44	Fever, blurry vision, change in mental status MRI showed a 4.7cm sellar mass with suprasellar extension and chiasma compression. Secondary adrenal insufficiency and hypothyroidism	High dose steroid therapy Transsphenoidal resection of pituitary adenoma which contains focal necrosis and hemorrhage	Recovery with thyroid and steroid hormonal deficits

Supplementary Table S1 (continued): Main characteristics of the case-report studies on pituitary adverse events following COVID-19 vaccines included in this review.

Adverse effect	Author (Ref)	Vaccine type	Latency between vaccine (dose) and symptoms (days)	Sex (M/F)	Age (years)	Clinical presentation	Treatment	Outcome
Arginine vasopressin deficiency (Central diabetes insipidus)	Ishay et al. [7]	mRNA based BNT162b2	60 days after the 1st dose	F	59	Polyuria polydipsia hypernatremia<br>Low urine and high serum osmolarity<br>Water deprivation test confirm central diabetes insipidus<br>MRI showed thickened pituitary stalk without bright spot of neurohypophysis<br>Anterior pituitary function was normal	Oral desmopressin	At 2 years follow-up still need for desmopressin and no change on MRI findings
Arginine vasopressin deficiency (Central diabetes insipidus)	Bouça et al [97]	mRNA based BNT162b2	7days after the 2nd dose	F	37	Intense thirst, polyuria >10l/24h<br>Urine osmolality 75mOsm/kg<br>MRI: loss of posterior pituitary bright spot<br>Normal anterior pituitary function	Oral desmopressin	At six-month follow-up no polyuria or polydipsia under oral desmopressin treatment

Supplementary Table S1 (continued): Main characteristics of the case-report studies on pituitary adverse events following COVID-19 vaccines included in this review.

Adverse effect	Author (Ref)	Vaccine type	Latency between vaccine (dose) and symptoms (days)	Sex (M/F)	Age (years)	Clinical presentation	Treatment	Outcome
Hypophysitis with Arginine vasopressin deficiency (Central diabetes insipidus)	Ach et al [98]	viral vector based Oxford-AstraZeneca ChAdOx1	3 days after the 1st dose	F	55	Polyuria polydipsia, fatigue hypernatremia low urine and high serum osmolarity. Water deprivation test confirm the diagnosis of central diabetes insipidus. MRI showed thickening of the pituitary stalk suggestive of infundibuloneurohypophysitis Anterior pituitary function was normal	Oral desmopressin	Remission with oral desmopressin
Arginine vasopressin deficiency (Central diabetes insipidus)	Partenope et al [99]	mRNA based BNT162b2	18 days after the 1st dose	M	16	Polyuria polydypsia Weight loss Normal glucose level. Borderline high serum sodium. Normal blood osmolarity very low urine osmolarity Ex juvantibus diagnosis of central diabetes insipidus was made after administration of desmopressin Anterior pituitary function was normal MRI showed thickened pituitary stalk without bright spot of neurohypophysis	Oral desmopressin	At 2 months no change in MRI findings and still need for desmopressin treatment

Supplementary Table S1 (continued): Main characteristics of the case-report studies on pituitary adverse events following COVID-19 vaccines included in this review.

Adverse effect	Author (Ref)	Vaccine type	Latency between vaccine (dose) and symptoms (days)	Sex (M/F)	Age (years)	Clinical presentation	Treatment	Outcome
Arginine vasopressin deficiency (Central diabetes insipidus) and hypophysitis with bilateral optic neuritis	Matsuo et al [100]	Moderna mRNA Spikevax (elasomeran)	30 days after the 4th dose	F	74	Increased thirst, polyuria, polydipsia<br>Low plasma vasopressin. Low urine osmolarity with >3-fold increase after desmopressin test<br>Normal pituitary anterior function<br>MRI: thickening of pituitary stalk, absence of posterior pituitary spot. Mild increase size of pituitary gland<br>High enhancement of the whole pituitary with gadolinium	Oral desmopressin<br>Steroid pulse therapy	At two months, oral desmopressin successfully alleviated diabetes insipidus. However, she was diagnosed with bilateral optic neuritis linked to hypophysitis and spastic hemiplegia with gait disturbances as manifestation of concomitant multiple sclerosis
Hypophysitis	Murvelashvili et al [101]	Moderna mRNA - 1273	3 days after the first dose<br>2 days after the second dose	M	51	Nausea, vomiting, abdominal pain<br>Severe hyponatremia central hypothyroidism and hypogonadism<br>Undetectable cortisol and low ACTH levels<br>Enlargement of pituitary gland with thickening of pituitary stalk	High dose steroids and thyroid hormone replacement therapy	After one month markedly reduction in enlargement of hypophysis size with mostly empty sella.<br>Normalization of testosterone level<br>Discharged with steroids and thyroxin replacement therapy

Supplementary Table S1 (continued): Main characteristics of the case-report studies on pituitary adverse events following COVID-19 vaccines included in this review.

Adverse effect	Author (Ref)	Vaccine type	Latency between vaccine (dose) and symptoms (days)	Sex (M/F)	Age (years)	Clinical presentation	Treatment	Outcome
Hypophysitis with Arginine vasopressin deficiency (Central diabetes insipidus)	Ankireddypalli et al [102]	mRNA based BNT162b2	2 days after the 1st dose	F	48	Polyuria polydipsia lethargy headache<br>Transient amenorrhea<br>MRI showed thickened pituitary stalk and partial empty sella<br>Endocrine pituitary adrenal and thyroid axis were normal<br>Low urine and high serum osmolarity. Water deprivation test confirms the diagnosis of central diabetes insipidus	Intranasal desmopressin therapy	At 5 months after vaccination still need for desmopressin treatment<br>Amenorrhea resolved<br>No change in MRI findings
Isolated ACTH deficiency	Morita et al [103]	mRNA based BNT162b2	1 day after the 2nd dose	M	31	General fatigue, Fever, headache nausea diarrhea low BP hyponatremia. Very low ACTH and cortisol levels<br>MRI showed moderate atrophy of the anterior pituitary<br>No other anterior pituitary hormones deficiency<br>No AVP deficiency	Hydrocortisone	At 3 months recovery with ongoing hydrocortisone therapy
Syndrome of inappropriate antidiuresis (SIADH)	Lindner et al [104]	Moderna 1273-mRNA	9 days after the 2nd dose	M	79	Weakness, anorexia<br>Severe hyponatremia, high urine osmolarity and sodium<br>Normal basal cortisol	Fluid restriction<br>Intravenous crystalloid solutions<br>Oral urea	Marked improvement<br>Within 7 days the serum sodium was normalized
