# Supplementary material for: SARS-CoV-2-Vaccine-Related Endocrine Disorders: An Updated Narrative Review
Source: Vaccines (Basel). 2024 Jul 8;12(7):750. doi: 10.3390/vaccines12070750 (PMC11281608; doi:10.3390/vaccines12070750)
Supplement: Supplementary file 1 [file vaccines-12-00750-s001.zip › Table S2.pdf]

Supplementary Table S2: Summarizing data regarding adrenal adverse events (other than hemorrhage/infarction) after COVID-19 vaccine.

**Author [Ref]**	**Vaccine type**	**Sex/age (years)**	**Latency between vaccine (dose) and symptoms**	**Medical history**	**Clinical Presentation/Diagnosis**	**Treatment**	**Outcome**
Maguire D at al Case 1 [115]	Viral vector based ChAdOx1	F/66	1 day after 1st dose	Addison's disease	Adrenal crisis	Stress doses of glucocorticoids	Discharged with double dose of glucocorticoids
Maguire D at al Case 2 [115]	Viral vector based ChAdOx1	M/ age not reported	~ 7 hours after 1st dose	Addison's disease<br>Short bowel syndrome secondary to mesenteric panniculitis	Adrenal crisis	Stress doses of glucocorticoids<br>IV fluids	Discharged within few days with his standard doses of hydrocortisone and fludrocortisone
Maguire D at al Case 3 [115]	Viral vector based ChAdOx1	F/69	1 day after 1st dose	Autoimmune polyglandular syndrome type 2 (including Addison's disease)	Adrenal crisis	Stress doses of glucocorticoids	Improvement. After another 2 days of two-fold increase in her standard dose of glucocorticoid, loss of follow up.
Maguire D at al Case 4 [115]	Viral vector based ChAdOx1	F/41	~9 hours after 1st dose	Panhypopituitarism after transsphenoidal surgery for Cushing disease	Adrenal crisis	Stress doses of glucocorticoids<br>IV fluids	Discharged within 24 hours
Maguire D at al Case 5 [115]	Viral vector based ChAdOx1	M/74	~5 hours after 1st dose	Panhypopituitarism after transsphenoidal surgery for non-functioning pituitary adenoma	Adrenal crisis	Stress doses of glucocorticoids	Recovery. Keeping on two-fold increase in standard dose of glucocorticoids for 3 days
Markovic N et al [120]	mRNA based BNT162b2	M/74	Few hours after 2nd dose	Panhypopituitarism after transsphenoidal surgery for prolactinoma	Adrenal crisis	Stress doses of glucocorticoids	Discharged within 24 hours with instructions for tapering glucocorticoids dose

Supplementary Table S2 (continued). Summarizing data regarding adrenal adverse events (other than hemorrhage/infarction) after COVID-19 vaccine.

**Author [Ref]**	**Vaccine type**	**Sex/age (years)**	**Latency between vaccine (dose) and symptoms**	**Medical history**	**Clinical Presentation/Diagnosis**	**Treatment**	**Outcome**
Haji Jr N et al [119]	Viral vector Johnson& Johnson COVID-19 vaccine	M/63	1 day, dose not reported	daily marijuana use	Pheochromocytoma multisystem crisis due to a 7cm right adrenal tumor, including non-ST-elevation myocardial infarction (NSTEMI) with cardiogenic shock, non-sustained ventricular tachycardia, and multiple upper and lower extremity deep vein thromboses	Supportive cardiopulmonary measures for stabilization and subsequent open adrenalectomy	Recovery within 3 months
