# Supplementary material for: SARS-CoV-2-Vaccine-Related Endocrine Disorders: An Updated Narrative Review
Source: Vaccines (Basel). 2024 Jul 8;12(7):750. doi: 10.3390/vaccines12070750 (PMC11281608; doi:10.3390/vaccines12070750)
Supplement: Supplementary file 1 [file vaccines-12-00750-s001.zip › Table S3.pdf]

Supplementary Table S3: Summarized data from case reports regarding COVID-19 vaccine related adrenal hemorrhage (AH) or infarction (AI).

Author [Ref]	Vaccine type	Sex/age (years)	Latency between vaccine (dose) and symptoms (days)	Unilateral or bilateral AH/AI	Risk factors	Radiological characteristics	Extra-adrenal thrombosis	Outcome
Taylor P [121]	Viral vector based ChAdOx1	M/38	8 days after 1st dose	Bilateral adrenal hemorrhage in the context of definite VITT	None	Retroperitoneal fat stranding, high density fluid around adrenals	Yes (PE, CVST)	Recovery with long term primary adrenal insufficiency
VaronaJF [122]	Viral vector based ChAdOx1	M/47	10 days, dose not reported	Bilateral adrenal hemorrhage in the context of definite VITT	None	Subacute bilateral adrenal hemorrhage	Yes (PE, CVST)	Recovery with long term primary adrenal insufficiency
Tews H [123]	Viral vector based AD26.COV2.S	M/39	10 days after 1st dose	Bilateral adrenal hemorrhage in the context of definite VITT	None	Acute bilateral adrenal hemorrhage	Yes (PE)	Recovery with long term primary adrenal insufficiency
Blauenfeldt RA [124]	Viral vector based ChAdOx1	F/60	7 days after 1st dose	Bilateral adrenal hemorrhage in the context of definite VITT	Hypertension	Acute bilateral adrenal hemorrhage	Yes (ischemic stroke)	Death on the sixth hospital day
D'Agostino V [125]	Viral vector based ChAdOx1	F/54	12 days, dose not reported	Bilateral adrenal hemorrhage in the context of definite VITT	None	Acute bilateral adrenal hemorrhage	Yes (extensive right coronary and pulmonary arteries thrombosis)	Death on the fifth hospital day
Al Rawahi B [126]	Viral vector based ChAdOx1	M/64	7 days after 1st dose	Bilateral adrenal hemorrhage in the context of definite VITT	Hypertension Hyperlipidemia	Acute bilateral adrenal hemorrhage	Yes (Multiple sites of venous and arterial thrombosis: pulmonary, adrenal, renal, aorta)	Recovery with long term primary adrenal insufficiency
Graf A case 1 [127]	Viral vector based ChAdOx1	M/46	8 days after 1st dose	Bilateral adrenal hemorrhage in the context of definite VITT	None	Right AH identified day 1 Left AH identified day 4	Yes (PE, CSVD, portal and hepatic vein thrombosis, bilateral renal cortical infarcts Occipital lobe brain infarction, AMI)	Recovery with long term primary adrenal insufficiency

Abbreviations: PE, pulmonary embolism; CVST, cerebral venous sinus thrombosis; VITT, vaccine-induced immune thrombotic thrombocytopenia; AMI, Acute myocardial infarction

Supplementary Table S3 (continued): Summarized data from case reports regarding COVID-19 vaccine related adrenal hemorrhage (AH) or infarction (AI).

Author [Ref]	Vaccine type	Sex/age (years)	Latency between vaccine (dose) and symptoms (days)	Unilateral or bilateral AH/AI	Risk factors	Radiological characteristics	Extra-adrenal thrombosis	Outcome
Graf A case 2 [127]	Viral vector based ChAdOx1	F/38	11 days after 1st dose	Unilateral adrenal infarction	Obesity, spondyloepiphyseal dysplasia, osteoarthritis	enlarged left adrenal gland, with loss of shape and decreased attenuation compatible with evolving infarction and post-infarction necrosis	Yes, left renal vein thrombosis	Discharge with empirical oral hydrocortisone therapy
Efthymiadis A et al [128]	Viral vector based ChAdOx1	F/23	8 days after 1st dose	Bilateral adrenal hemorrhage in the context of definite VITT	Obesity, family history of venous thromboembolism	Bilateral ill-defined lesions representing bilateral adrenal hemorrhages	Yes, multiple pulmonary emboli, bilateral adrenal vein thrombosis, splenic vein thrombosis and right ventricular thrombosis	Resolution of adrenal hemorrhage. Resolution of pulmonary and right ventricle thrombosis. Persistent primary adrenal insufficiency
Tha T et al [129]	Viral vector based ChAdOx1	F/47	8 days, dose not reported	Bilateral adrenal hemorrhage in the context of definite VITT	None	Bilateral adrenal enlargement with high attenuation density measurement characteristic of AH	Yes, bilateral adrenal vein thrombosis, right renal vein thrombosis with renal infarction and pulmonary embolism	Discharged with replacement therapy for confirmed primary adrenal insufficiency
Douxfils J et al [130]	Viral vector based ChAdOx1	F/83	14 days after the 1st dose	Right adrenal hematoma and left adrenal infarction in the context of VITT	Chronic obstructive pulmonary disease	Right adrenal hematoma and left adrenal infiltrate compatible with AI	Bilateral pulmonary embolism	Death at day 14 from hypovolemic shock probably secondary to adrenal hemorrhage

Abbreviations: PE, pulmonary embolism; CVST, cerebral venous sinus thrombosis; VITT, vaccine-induced immune thrombotic thrombocytopenia; AMI, Acute myocardial infarction

Supplementary Table S3 (continued): Summarized data from case reports regarding COVID-19 vaccine related adrenal hemorrhage (AH) or infarction (AI).

Author [Ref]	Vaccine type	Sex/age (years)	Latency between vaccine (dose) and symptoms (days)	Unilateral or bilateral AH/AI	Risk factors	Radiological characteristics	Extra-adrenal thrombosis	Outcome
Boyle L et al [131]	Viral vector based ChAdOx1	F/55	8 days after 1st dose	Unilateral AH	Hypertension	Left adrenal enlargement suggestive of hemorrhage	Yes (lungs, ovarian vein, basilic vein)	Recovery with replacement therapy for confirmed adrenal insufficiency
Ahmad A et al. [132]	Viral vector based ChAdOx1	F/23	10 days after 1st dose	Bilateral adrenal hemorrhage in the context of probable VITT	Anticoagulant use	Bilateral adrenal hemorrhage with fat stranding	Yes, (CVST, splenic vein, right ventricle)	Discharged with replacement therapy for confirmed adrenal insufficiency

Abbreviations: PE, pulmonary embolism; CVST, cerebral venous sinus thrombosis; VITT, vaccine-induced immune thrombotic thrombocytopenia; AMI, Acute myocardial infarction
